# HeMoQuest: a webserver for qualitative prediction of transient heme binding to protein motifs

**DOI:** 10.1186/s12859-020-3420-2

**Published:** 2020-03-27

**Authors:** Ajay Abisheck Paul George, Mauricio Lacerda, Benjamin Franz Syllwasschy, Marie-Thérèse Hopp, Amelie Wißbrock, Diana Imhof

**Affiliations:** Pharmaceutical Biochemistry and Bioanalytics, Pharmaceutical Institute, An der Immenburg 4, University of Bonn, 53121 Bonn, Germany

**Keywords:** Heme, Heme-regulated protein, Transient heme binding, Heme-binding site prediction, Web application, Machine learning

## Abstract

**Background:**

The notion of heme as a regulator of many physiological processes via transient binding to proteins is one that is recently being acknowledged. The broad spectrum of the effects of heme makes it important to identify further heme-regulated proteins to understand physiological and pathological processes. Moreover, several proteins were shown to be functionally regulated by interaction with heme, yet, for some of them the heme-binding site(s) remain unknown. The presented application HeMoQuest enables identification and qualitative evaluation of such heme-binding motifs from protein sequences.

**Results:**

We present HeMoQuest, an online interface (http://bit.ly/hemoquest) to algorithms that provide the user with two distinct qualitative benefits. First, our implementation rapidly detects transient heme binding to nonapeptide motifs from protein sequences provided as input. Additionally, the potential of each predicted motif to bind heme is qualitatively gauged by assigning binding affinities predicted by an ensemble learning implementation, trained on experimentally determined binding affinity data. Extensive testing of our implementation on both existing and new manually curated datasets reveal that our method produces an unprecedented level of accuracy (92%) in identifying those residues assigned “heme binding” in all of the datasets used. Next, the machine learning implementation for the prediction and qualitative assignment of binding affinities to the predicted motifs achieved 71% accuracy on our data.

**Conclusions:**

Heme plays a crucial role as a regulatory molecule exerting functional consequences via transient binding to surfaces of target proteins. HeMoQuest is designed to address this imperative need for a computational approach that enables rapid detection of heme-binding motifs from protein datasets. While most existing implementations attempt to predict sites of permanent heme binding, this application is to the best of our knowledge, the first of its kind to address the significance of predicting transient heme binding to proteins.

## Background

Heme (iron protoporphyrin IX) is an astoundingly prevalent molecule found within humans, animals and plants, fulfilling a plethora of functions [1, 2]. It is the oxygen carrying moiety of hemoglobin, the gas-sensing molecule of NO-sensors and the redox active part of cytochromes [1, 2]. Besides its well-known binding to these hemoproteins as a prosthetic group, heme has been established as a biologically available molecule. Human targets of transient heme binding have been reviewed extensively [1–6] (Additional Table 1, supplementary data 3). Well-known representatives are δ-aminolevulinic acid synthase 1 (ALAS1) and transcription regulator protein Bach1, which bind heme via CP-containing motifs [1, 7, 8]. Recent reports have expanded the knowledge on transient heme binding. Some of the published heme-regulated proteins were shown to bind heme, but no information on the heme binding motif is available so far. A curated list of such proteins is available in additional Table 1. In this work we highlight the need for an exclusive computational method that is able to pinpoint heme-binding residues in protein sequences.

**Fig. 1:**
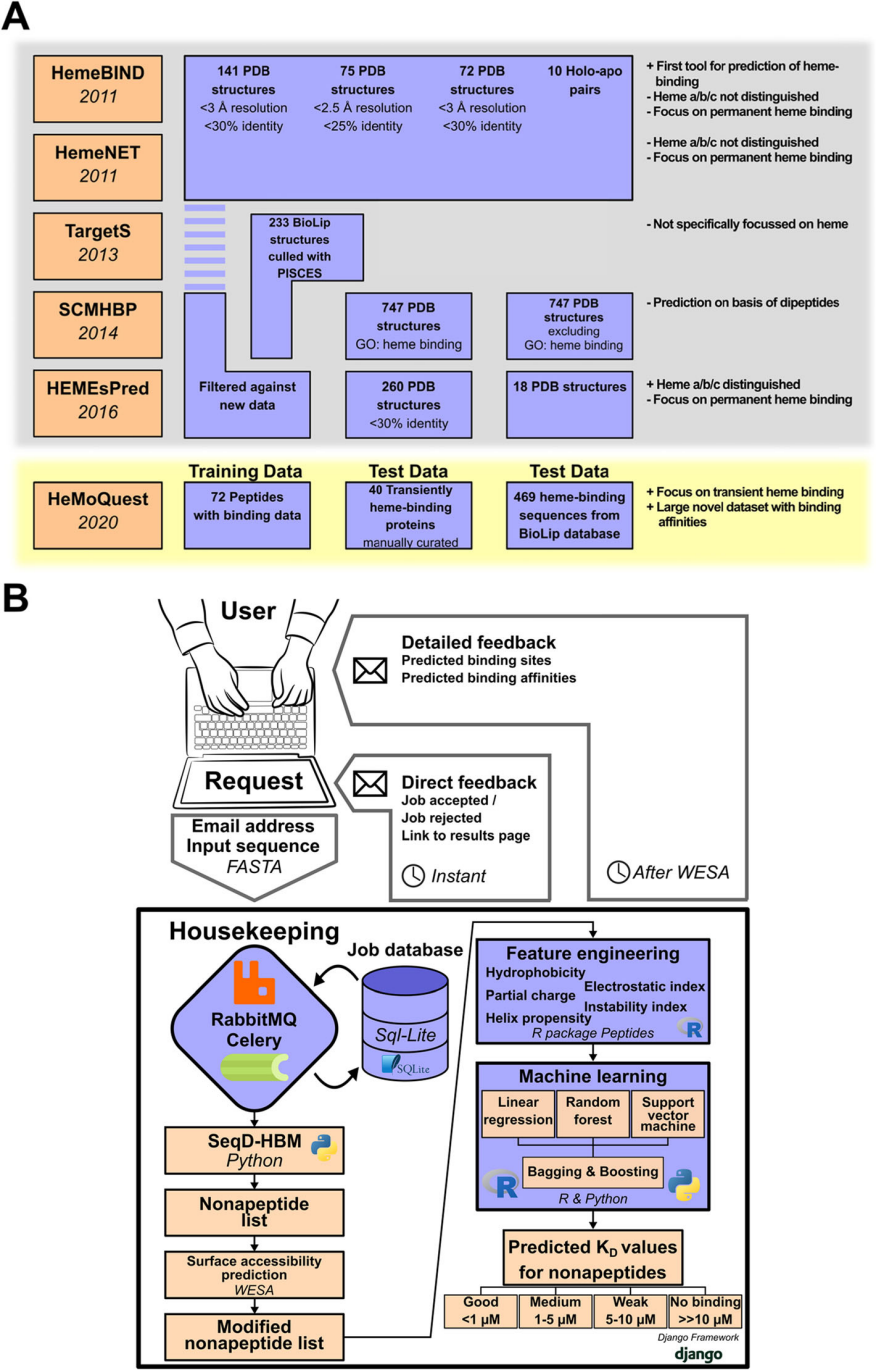
**a** Datasets used in previous publications and their respective cutoff values. **b** Schematic representation of the HeMoQuest web application architecture

Despite the apparent abundance of interest in computational solutions to predict heme binding, all of the former approaches were focused on prediction of permanent heme binding as opposed to the prediction of transient heme binding and its associated regulatory function. In 2011 and 2012, the groups of Liu, Li and Xiong were the first to present publicly available heme-binding prediction algorithms [9–12]. These approaches had in common that they started from sets of published structures of hemoproteins. A large number of such structures is available, i.e. the gene ontology term “heme binding” (GO:0020037) currently yields over 4500 PDB structures. However, these structures are highly redundant and some are of low resolution, which was compensated by the authors by applying a cutoff at 25% or 30% sequence identity and at 3 Å resolution [9–12]. The resulting datasets were used to train machine learning algorithms based on structural features. In 2013, Yu et al. took the challenge to predict heme binding to proteins without available 3D structures, since all the so far known webservers were working with template-based methods. Therefore, the webserver “TargetS” (http://www.csbio.sjtu.edu.cn/ bioinf/TargetS/) was established to predict binding of ligands (i.a. heme) starting from primary sequences via a recursive spatial clustering algorithm. It included different aspects, such as evolutionary information, ligand-specific properties and secondary structure. A dataset of 233 structures of heme-binding proteins with a cutoff of 40% sequence identity was extracted from the BioLIP database [13], and used for training and testing the webserver. Derived from a scoring card method (SCM), the latest prediction method for heme binding to proteins “SCMHBP” benefits from an evaluation of heme-binding tendencies of 400 dipeptides and 20 amino acids, which is transferred onto protein sequences. Consequently, two non-redundant training datasets were designed with 747 heme-binding proteins and 747 non-heme-binding proteins, and two already existing datasets were taken into account for testing the SCMHBP, resulting in a mean accuracy of 85.9% [9, 10, 14]. In another approach, Zhang et al. clustered 4003 X-ray structures of heme-binding proteins via Blastclust [15] with a sequence identity of less than 30% and selected 260 representatives for testing [16]. In addition, the training datasets from earlier studies were included [10, 17]. On this data, a novel predictor, i.e. “HEMEsPred” (http://www.inforstation.com/HEMEsPred/), was generated including sequence- and structure-based features, a fast-adaptive ensemble learning scheme and a more specific model for different heme ligands [16]. A summary of previous algorithms can be found in Fig. 1a.

However, all of these approaches employed relatively generic training data by merely querying available databases. Large parts of these training data consist of biologically redundant data such as different variants of hemoglobins (29.2%), and cytochromes (34.8%). Furthermore, previous studies were entirely focused on permanent heme binding and thus, disregarded transient, regulatory heme binding entirely. In contrast to hemoproteins, transient heme binding is believed to occur on protein surfaces and not in deep heme-binding pockets [1, 2, 6]. Strikingly, previous implementations have strongly focused on residues in the protein core, at times even specifically excluding surface exposed residues. As a consequence, all previous algorithms fail to predict transient heme-binding sites, and attain a maximum accuracy of less than 60% when challenged with the prediction of transient heme binding. The overarching aim of this work is to provide a computational tool exclusively developed for the prediction and qualitative evaluation of transient heme binding to protein surfaces based on our recently established SeqD-HBM algorithm [6].

## Results

The first version of the algorithm was produced as a standalone Python script(s) wherein the SeqD-HBM algorithm for detecting heme-binding motifs was implemented only [6]. At this stage, the program was used as an in-house tool and was shared with users upon request. The full-fledged web application, in contrast, is a multi-fold improvement on the former version. It is built on the Django 2.3 framework (https://www.djangoproject.com/), which is known for its robustness, scalability and security. As shown in Fig. 1b, the usage of RabbitMQ and Celery for the management of user requests in the housekeeping module makes it evident that the application was built to scale well on heavy load. This is especially important since the application handles not only inbound and outbound user requests but also makes API calls to the (external) weighted ensemble solvent accessibility (WESA) [18, 19] application in the process of providing predictions. That being the case, additional measures had to be taken to constantly check all of the application end-points and update the status of a user request in the database. HeMoQuest was able to correctly predict residues (and the associated motifs) annotated as “heme-binding” with accuracies of 92.1% while using the WESA mode and 96.2% when WESA solvent accessibility was skipped from the 469 sequences gathered from BioLip (Supplementary data 2). In the WESA mode a small dip in accuracy was due to the fact that most CP motifs were predicted to be buried by WESA. At the same time, though the non-WESA mode of operation an increase in accuracy was produced, the number of new potential motifs predicted increased by ~ 15%. However, it must be noted that these additionally predicted motifs cannot be deemed as false positives with certainty since there exists no experimental evidence to prove their inability to transiently bind heme. Going beyond detecting heme-binding motifs from input sequences, the fact that a reasonable amount of in-house data with published binding affinities existed (Additional Table 2, supplementary data 1), provided the scope for a predictive machine-learning based solution to be implemented. In essence, the idea is to train the sequence data with known affinities on different predictive models so that a new input sequence could have the approximate binding affinities of their nonapeptide motifs predicted. We explicitly state here that the predicted affinities are not to be compared with the experimental values published at the protein level but are only to be compared among the different nonapeptides from the same sequence as an indicator of the best binding motif in the input sequence. Features from the input sequences were engineered by using the *Peptides* R package (https://github.com/dosorio/Peptides/). For any input sequence *Peptides* can produce over 100 different physiochemical values that define features such as hydrophobicity, instability, partial charge etc. (Supplementary data 4). It is infeasible to train a model on such a large number of features and hence the feature engineering consisted of choosing a reasonable number of diverse features that describe the input sequence. The Pearson correlation coefficient was calculated between all physiochemical features available in *Peptides* on the binding affinity data and the top 8 most diverse features that produced the best correlation were chosen as the final features for predictions. These features, which described hydrophobicity, instability, helix propensity, partial charge and electrostatics, were all sequence invariants, i.e. they are strongly dependent on the order of the amino acid residues in the sequence. All of the machine learning models were built using the Scikit-learn machine learning package [20].

Since the aim was to predict binding affinities, regression was used in all models built. First, a multivariate linear regression model was built using the Ordinary Least Squares method. Next, a random forest regressor, which is a meta estimator that fits a number of classifying decision trees on various sub-samples of the dataset and uses averaging to improve the predictive accuracy and control over-fitting, was built. Finally, a regression model of a SVM was built using the Epsilon-Support Vector Regression method available in Scikit-learn. Predictions from the individual models were further subjected to an independent voting scheme to produce the best prediction. The AdaBoost and BaggingRegressor methods were used for the purpose, respectively.

The application was tested on the three independently collected datasets (see Methods). Overall, the heme-binding residue prediction module was able to successfully identify the predefined heme-coordinating residues and the associated motifs. Our method was able to successfully predict every single heme-coordinating residue and its associated motif for every single sequence tested. This is mainly due to the specific checks done on H, C, and Y residues explicitly on all the sequences. Consequently, the algorithm predicted more motifs than what is mentioned in the validation set. Though this results in an overall false positive rate of ~ 15%, one cannot be sure that these “additional” motifs are other potential heme-binding motifs, not accessible for prediction via experimental approaches as seen in earlier reports [21]. The prediction of affinities was again impressive with the support vector machine producing the best predictions of 71% accuracy on the training set. In terms of the qualitative classification, we were able to correctly classify with an overall accuracy of 68%, between the “good”, “moderate”, and “weak” binding motifs on the test set.

It was further observed that within the individual prediction algorithms (Fig. 1a), the linear regressor preformed the worst since it was clear that none of the physiochemical features used to describe the sequence data had strong correlation to the K_D_ values. This is a common pitfall for linearly uncorrelated features and is frequent in small datasets. However, the random forest and SVM predictions outweighed poor performance of the linear regression, thereby producing overall predictions of acceptable quality.

## Discussion

Under hemolytic conditions, such as thalassemia, sickle cell disease, or distinct bacterial infections, red blood cells are destroyed and release both hemoglobin and heme [3]. Heme is initially bound and neutralized by heme scavengers such as hemopexin and albumin, but once their capacity is exceeded, vast amounts of free heme arise [22]. Heme can consequently bind and regulate a number of proteins, for most of which the interaction site is unknown. For example, heme has been suggested to inhibit the classical complement pathway by interaction with C1q [23] and to activate the alternative complement pathway by deposition of C3 [24]. Using Surface Plasmon Resonance spectroscopy, the heme dissociation constant of C1q was determined to be approximately 1–2 μM, but no binding site could be identified yet [25]. The latter applies to C3 as well, yet molecular docking suggested a binding site close to the functionally important thioester bond [24]. Furthermore, heme influences factor VIII and fibrinogen in seemingly contradictory fashion, but partially due to the lack of structural information this dissonance has not been unraveled yet [26–28].

The opposite approach, i.e. the prediction of unknown heme-regulated proteins from peptide sequences, has also been fruitful. Building on a combinatorial peptide library screening approach we predicted and validated transient heme binding to proteins such as chloramphenicol acetyltransferase, hemolysin C, and interleukin-36α [6, 29–31].

## Conclusion

As demonstrated with evidence thus far, heme plays a key regulatory role in a multitude of processes. Strikingly, for most of the proteins involved, little is known about the sites and affinities of heme binding. Experimental mapping of heme-protein interactions requires vast effort, e.g. the expression of protein mutants or the co-crystallization of heme with the protein of interest, at times with conflicting results [8, 32]. As an extension of existing experimental work on peptide-heme binding, HeMoQuest provides a shortcut to the identification of heme-regulated proteins. Due to the convincing accuracy of the presented algorithm, researchers may be able to bypass the necessity of producing peptides as models and may even be able to rationally design heme-binding peptides and proteins. Several efforts have been undertaken to predict heme binding to proteins [9–12, 14, 16, 17, 33], however, HeMoQuest is fundamentally different from previous tools because of its exclusive focus on transient heme binding. The tool is built on a dataset created solely for this purpose. As with any data-driven approach, HeMoQuest is poised to only get better with time as more data becomes available.

## Methods

### Web application architecture

Multiple programming languages and frameworks were effectively utilized in this work to construct a user-friendly and effective web application, called HeMoQuest (heme-binding motif quest). This webserver (Fig. 1b) was built on the Django framework version 2.3.1 (https://www.djangoproject.com/) running Python 3.6.5 under the hood. The user is given access to three pages: 1) the landing page that can also be used to submit sequences for analysis, 2) the results page containing the analysis of a single request in a tabular format, and 3) the analysis page hyperlinked from the results providing a stepwise analysis of how a prediction was produced for an input sequence. User input and the analysis results are saved in a SQlite database (https://www.sqlite.org/index.html). The schema consists of three tables namely: ‘jobs’, ‘sequences’ and ‘results’. The job is saved whenever the user sends enough information to be processed, such as a file or an input sequence. The sequences table stores for each job all sequences successfully read (even if there are false inputs). The results table contains the predicted nonapeptide motifs and the predicted binding affinities.

### Web application control flow

Django first checks user submissions for the following: There must be at least one sequence or one file in the FASTA format. File sizes are restricted to 2 MB. If solvent accessibility prediction via WESA, i.e. the Weighted Ensemble Solvent Accessibility predictor tool [18, 19], is requested, an email address is required to be entered by the user. With the basic checks done, the input is read and a status (either “failed”, “queued” or “processed”) is assigned. Each sequence is analyzed and the possible binding sites are saved in the database. If solvent accessibility prediction was not requested, the analysis for each sequence is generated and saved, the status is changed to “processed”. If an email address was supplied, a message is sent with the link to the results page and finally the user is redirected to the results page. If solvent accessibility prediction was requested, the analysis for each sequence is also generated and saved, under the status “queued”. The user will receive an email with uniform resource locator (URL) link to the results page that displays the status of how many sequences are being processed and, as the sequences are processed, their results. This process will continue as long as the celery job detects the existence of queued sequences with the WESA detection mode. The task scheduler takes care of the automatic execution of this process.

### Prediction algorithms

The nonapeptide motif prediction algorithm SeqD-HBM published earlier [6] as a standalone python script was overhauled and rewritten for the web application. For the prediction of binding affinities, a set of 73 nonapeptide sequences (Additional Table 1) synthesized and validated in-house with experimentally determined and published binding affinities were used [29, 30, 34–36]. Three different predictors namely, linear regression, random forest and support vector machine (SVM) methods were used to perform regression, predicting the target variable, i.e. the binding affinity. An ensemble-based voting scheme was used to obtain the final prediction from the individual predictors (Fig. 1b). An 80/20 train-test split was employed in all cases.

### Dataset preparation

Three independent datasets were used for this study. The first of which comprised of 469 sequences (supplementary data 2) from the BioLip database [13] (March 2019 release) extracted for the HEM ligand code which relates to “heme”. The second dataset consisted of a cumulative set of all of the data used in previous studies (Fig. 1b). Finally, a set of 40 proteins (Additional Table 2, supplementary data 3) known to bind heme transiently, was chosen manually.

## Supplementary information

**Additional file 1:.** Additional Table 1. Human heme-regulated proteins and reported heme-binding sites. Additional Table 2. Peptides sequences and binding data used for the initial training of HeMoQuest

**Additional file 2 **Supplementary data Additionally, the datasets used to train and validate the application is also available for download from the “HeMoQuest Datasets” section of the webserver. A description of the files provided is given below. 1. Training data. 1a. Title: HeMoQuest K_D_ prediction training data. 1b. Description: This comma separated values file contains 72 sequences along with their K_D_ values used in the training of the ML algorithms of HeMoQuest. Column 1 (ID) contains a sequence identifier, column 2 (Seq) contains the sequence and column 3 (K_D_) contains the experimentally determined K_D_ value of for the peptide sequence. 2. Test data. 2a. Title: HeMoQuest test data for heme binding residue and motif prediction. 2b. Description: This file contains 469 sequences in fasta format, obtained from the BioLip database, all of which are said to bind heme. This data was used to test HeMoQuest’s ability to detect heme binding residues in comparison to existing algorithms. 3. Test data. 3a. Title: HemoQuest test data with manually curated transient heme binding protein sequences. 3b. Description: This file contains 45 sequences in fasta format from 40 manually curated proteins (from Additional Table 1) that are known from literature to be transient heme binding proteins. Few of the proteins have their origins in more than one species and hence we end up with 45 sequences for 40 proteins. 4. Training features. 4a. Title: Features used in training the HeMoQuest K_D_ prediction. 4b. Description: This comma separated values file contains 76 initial features that were generated for the K_D_ prediction training from the R package *Peptides*. The final set of features used are from the columns ‘charge_vec’, ‘hydrof_vec_octanolScale_pH8’, ‘acidic’, ‘kideraFac3’, ‘vhseScale5_vec’, ‘vhseScale7_vec’, ‘protFP5_vec’ and ‘fasgaiVec4’.

## Data Availability

The HeMoQuest webserver is freely available at http://bit.ly/hemoquest All the data used in this study are available in additional files 1 and 2.

## Abbreviations

ALAS: Aminolevulinic acid synthase
NO: Nitric oxide
SeqD-HBM: Sequence based detection of heme-binding motifs
SVM: Support vector machine
URL: Uniform resource locator
WESA: Weighted ensemble solvent accessibility
