# Supplementary material for: HeMoQuest: a webserver for qualitative prediction of transient heme binding to protein motifs
Source: BMC Bioinformatics. 2020 Mar 27;21:124. doi: 10.1186/s12859-020-3420-2 (PMC7099796; doi:10.1186/s12859-020-3420-2)
Supplement: Supplementary file 1 — Additional file 1:. Additional Table 1. Human heme-regulated proteins and reported heme-binding sites. Additional Table 2. Peptides sequences and binding data used for the initial training of HeMoQuest [file 12859_2020_3420_MOESM1_ESM.docx]

HeMoQuest: A webserver for qualitative prediction of transient heme binding to protein motifs

Ajay Abisheck Paul George^1^, Mauricio Lacerda^1^, Benjamin Franz Syllwasschy^1^, Marie-Thérèse Hopp^1^, Amelie Wißbrock^1^, and Diana Imhof^1,*^

^1^Pharmaceutical Biochemistry and Bioanalytics, Pharmaceutical Institute, An der Immenburg 4, University of Bonn, 53121 Bonn, Germany

*To whom correspondence should be addressed: Diana Imhof (Email: dimhof@uni-bonn.de).

**Additional information**

Additional Table 1. Human heme-regulated proteins and reported heme-binding sites

| **Number** | **Protein** | **Origin** | **Published heme-binding sites** | **References** | **HeMoQuest**  **Prediction*** | **Comment** |
| --- | --- | --- | --- | --- | --- | --- |
| 1 | δ-aminolevulinic acid synthase 1 (ALAS1) | *R. norvegicus/ H. sapiens* | RC^8^PFLS  NC^33^PKMM  KC^108^PFLA | [1–3] | All published sites correctly predicted. | CP motifs missed in the WESA mode. |
| 2 | δ-aminolevulinic acid synthase 2 (ALAS2) | *M. musculus/ H. sapiens* | C^11^PVLA  C^38^PILA | [1, 4] | All published sites correctly predicted. | CP motifs missed in the WESA mode |
| 3 | Amyloid β | *H. sapiens/ rodents* | Y^10^EVH^13^H^14^ | [5–9] | Published site correctly predicted. | Used as training data. |
| 4 | Arginyl-Transferase (ATE1) | *M. musculus/ S. cerevisiae* | HSC^411^P | [10] | Published site correctly predicted. | CP motifs missed in the WESA mode. |
| 5 | Arginyl-tRNA-Synthetase (ArgRS) | *H. sapiens* | QC^115^NSAM | [11] | All published sites correctly predicted. | - |
| 6 | Bach1 | *M. musculus* | EC^438^PWLG  NC^464^PFIS  PCP^495^YAC  DC^649^PLSF | [12–15] | All published sites correctly predicted. | CP motifs missed in the WESA mode. |
| 7 | Bach2 | *H. sapiens* | C^369^P  C^499^P  C^506^P | [16–18] | All published sites correctly predicted. | CP motifs C^499^P  C^506^P missed and C^369^P predicted correctly in WESA mode. |
| 8 | BK channel | *H. sapiens* | C^612^XXCH^616^ | [19–23] | Published sites correctly predicted. | - |
| 9 | C1q | *H. sapiens* | not known | [24, 25] | Up to 6 potential motifs predicted | - |
| 10 | C3(a/b) | *H. sapiens* | not known | [26] | 20 potential motifs predicted | - |
| 11 | CLOCK | *M. musculus* | H^144^  C^195^ | [27] | All published sites correctly predicted. | - |
| 12 | Cystathionine-β-synthase (CBS) | *H. sapiens* | C^15^PHRSGPH  C^52^  H^65^ | [28–31] | All published sites correctly predicted. | - |
| 13 | DiGeorge critical region 8 (DGCR8) | *H. sapiens* | IPC^352^L (from two subunits) | [32–34] | All published sites correctly predicted. | - |
| 14 | Dipeptidyl peptidase 8 (DPP8) | *H. sapiens* | SDFKC^506^PIKE | [35] | All published sites correctly predicted. | - |
| 15 | Eukaryotic translation initiation factor 2a kinase 1 (eIF2α, HRI) | *M. musculus* | AC^167^PYVM  RC^309^PVQA | [36–38] | All published sites correctly predicted. | - |
| 16 | Anti-hemophilic factor (Factor VIII) | *H. sapiens* | not known | [39, 40] | 99 potential motifs predicted. | WESA could not be applied since the sequence is over 2000 residues. |
| 17 | Fibrinogen (Factor I) | *H. sapiens* | not known | [41, 42] | 12 potential motifs predicted. | - |
| 18 | Glyceraldehyde-3-phosphate dehydrogenase (GAPDH) | *H. sapiens* | not known | [43–47] | 3 potential motifs predicted. | - |
| 19 | Heme oxygenase 2 | *R. norvegicus/ H. sapiens* | XH^45^X  KC^265^PFYA (+H^256^)  SC^282^PFRT | [48–51] | All published sites correctly predicted. | - |
| 20 | Human period circadian protein homolog 2 (hPer2) | *H. sapiens* | SC^841^PA  AC^962^PA | [52] | All published sites correctly predicted. | - |
| 21 | Immunoglobin (IgG) | *H. sapiens* | not known | [53] | 8 potential sites predicted. | - |
| 22 | Interleukin-36α | *H. sapiens* | SEGGC^136^PLIL  FLFY^108^HSQSG | [54] | All published sites correctly predicted. | CP motifs missed by WESA mode. |
| 23 | Iron regulatory protein 1 (IRP1) | *H. sapiens* | C^118^P  C^300^P | [55, 56] | All published sites correctly predicted. | CP motifs missed by WESA mode. |
| 24 | Iron regulatory protein 2 (IRP2) | *H. sapiens* | C^201^PXH^204^XXXXP  C^375^P | [55–58] | All published sites correctly predicted. | CP motifs missed by WESA mode. |
| 25 | Janus kinase 2 (JAK2) | *H. sapiens* | RPDGC^1094^PDEI | [59] | All published sites correctly predicted. | CP motifs missed by WESA mode. |
| 26 | K_ATP_ channels | *R. norvegicus* | C^628^XXH(X)_16_H^648^ | [60] | All published sites correctly predicted. | - |
| 27 | Neudesin | *M. musculus* | not known | [61] | Up to 5 potential motifs predicted. | - |
| 28 | Neuferricin | *M. musculus* | not known | [62] | 6 potential motifs predicted. | - |
| 29 | NMDA Receptor | *M. musculus* | not known | [63, 64] | 33 potential motifs predicted. | - |
| 30 | p53 | *H. sapiens* | C^275^AC^277^P | [65, 66] | All published sites correctly predicted. | CP motifs missed by WESA mode. |
| 31 | p63 | *H. sapiens* | CXCP | [65] | All published sites correctly predicted. | CP motifs missed by WESA mode. |
| 32 | p73 | *H. sapiens* | CXCP | [65] | All published sites correctly predicted. | CP motifs missed by WESA mode. |
| 33 | PGRMC1/Sigma-2 receptor | *H. sapiens* | YGPEGPY^113^GVFA | [67, 68] | All published sites correctly predicted. | - |
| 34 | Rev-erbα | *H. sapiens* | H^602^ | [69–71] | All published sites correctly predicted. | - |
| 35 | Rev-erbβ | *H. sapiens* | C^384^  H^568^ | [70, 72–75] | All published sites correctly predicted. | - |
| 36 | Src | *H. sapiens* | not known (CP motif presumed) | [59] | Five potential motifs predicted. | Predicted CP motif C^486^P. |
| 37 | Stanniocalcin-1 | *H. sapiens* | RC^114^STFQ | [76] | All published sites correctly predicted. | - |
| 38 | Stanniocalcin-2 | *H. sapiens* | SRKC^125^PAIREM | [77] | All published sites correctly predicted. | - |
| 39 | Toll like Receptor 4 (TLR4) | *H. sapiens* | not known | [78] | 13 potential sites predicted. | - |
| 40 | Tryptophanyl-tRNA-Synthetase | *H. sapiens* | H^130^  (H^129^, H^336^, H^445^ presumed) | [79] | All published sites correctly predicted. | - |

*Sometimes, more potential binding sites than the reported ones were found.

Additional Table 2. Peptides sequences and binding data used for the initial training of HeMoQuest

| **Number** | **Sequence*** | **K_D_ [µM]** | **Reference** |
| --- | --- | --- | --- |
| 1 | RAFFYCKAC | 0.28 ± 0.09 | [80] |
| 2 | AFFYCKACH | 0.39 ± 0.09 | [80] |
| 3 | AESFCTNQD | 2.23 ± 1.86^†^ | [81] |
| 4 | KWEDCVFDS | 1.78 ± 0.78 | [81] |
| 5 | LWQFCAFSS | 0.79 ± 0.22 | [81] |
| 6 | WRSNCSHQH | 12.03 ± 0.59^†^ | [81] |
| 7 | VEKMCIETA | 5.56 ± 1.07 | [81] |
| 8 | AQEPHLEBK | 100 ± 0.00 | [82] |
| 9 | LINQHATSF | 6.23 ± 1.35 (n.sat.) | [82] |
| 10 | ETIFHTVQQ | 100 ± 0.00 | [82] |
| 11 | YPGQHISNE | 100 ± 0.00 | [82] |
| 12 | BVQLHKHSG | 2.41 ± 0.73 | [82] |
| 13 | RQRDHQBYA | 3.38 ± 0.68 | [82] |
| 14 | PRQAHVYRA | 4.78 ± 1.27 | [82] |
| 15 | IBFRYSSLK | 0.34 ± 0.26 | [82] |
| 16 | EKBWYWAEA | 100 ± 0.00 | [82] |
| 17 | EBRPYETGD | 100 ± 0.00 | [82] |
| 18 | LLPYYVQED | 100 ± 0.00 | [82] |
| 19 | GFGTYSWHE | 6.25 ± 1.44 | [82] |
| 20 | PPKTYQGEG | 100 ± 0.00 | [82] |
| 21 | DTPDYDYSR | 100 ± 0.00 | [82] |
| 22 | HADTYFGWR | 19.01 ± 1.76 (n.sat.) | [82] |
| 23 | ITSIYNGAQ | 100 ± 0.00 | [82] |
| 24 | LRAVYEKDA | 2.48 ± 0.48 (n.sat.) | [82] |
| 25 | FKQYHHELI | 2.31 ± 0.69^†^ | [82] |
| 26 | EQREHANVI | 100 ± 0.00 | [82] |
| 27 | HDENYTPPE | 100 ± 0.00 | [82] |
| 28 | KPFKYDHHY | 1.96 ± 0.72 | [82] |
| 29 | TLDLHLEVS | 100 ± 0.00 | [82] |
| 30 | AAAAAAAAA | 100 ± 0.00 | [9] |
| 31 | RHDSGYEVHH | 80.70 ± 4.00 | [9] |
| 32 | DAEFRHDSGYEVHHQKLV | 0.59 ± 0.05 | [9] |
| 33 | NVNLTSNALLYHYWIAVSHKAPA | 3.00 ± 1.19 | [9] |
| 34 | NVNLTSNHLLYHYWIAVSAKAPA | 1.89 ± 0.72 | [9] |
| 35 | NVNLTSNALLYHYWIAVSAKAPA | 0.88 ± 0.94 | [9] |
| 36 | NVNLTSNHLLYAYWIAVSHKAP | 19.84 ± 4.11 | [9] |
| 37 | DAEFRHDSGYEVHHQKLVFFAEDVGSNKGAIIGLMVGGVV | 0.10 ± 0.07 | [9] |
| 38 | AEFRHDSGY | 100 ± 0.00 | [9] |
| 39 | HDSGYEVHH | 100 ± 0.00 | [9] |
| 40 | RHLPCDICV | 0.13 ± 0.09 | [83] |
| 41 | FYWDCNHYW | 0.67 ± 0.12 | [80] |
| 42 | RADICVHLN | 0.40 ± 0.17 | [80] |
| 43 | SGGLPAPSDFKCPIKEEIAITSG | 1.42 ± 0.24 | [80] |
| 44 | AAAACAAAA | 100 ± 0.00 | [80] |
| 45 | QFSQCRIBN | 2.78 ± 0.78 | [80] |
| 46 | YVSRCIBBA | 3.44 ± 0.64 | [80] |
| 47 | HHQYHARVA | 0.87 ± 0.05 | [80] |
| 48 | AAAAHAAAA | 100 ± 0.00 | [80] |
| 49 | AAAAYAAAA | 100 ± 0.00 | [80] |
| 50 | HPFPYIWKA | 0.33 ± 0.25 | [80] |
| 51 | NVNLTSNHLLYHYWIAVSHKAPA | 1.40 ± 0.13 | [80] |
| 52 | VRMDTLAHVLYYPQKPLVTTRSM | 0.51 ± 0.27 | [80] |
| 53 | APSRCTQWL | 4.41 ± 0.80 | [35] |
| 54 | SSIPCLFYK | 0.28 ± 0.19 | [35] |
| 55 | SQSSCPAVP | 6.43 ± 0.53 | [35] |
| 56 | DESACPVYM | 13.26 ± 1.44 | [35] |
| 57 | RPDGCPDEI | 0.50 ± 0.23 | [35] |
| 58 | EDKDCPIKE | 48.40 ± 2.73 | [35] |
| 59 | ALTGCPWHD | 5.32 ± 0.55 | [35] |
| 60 | QFSSCPHYW | 1.94 ± 0.42 | [35] |
| 61 | IGVVCPFVR | 0.87 ± 0.41 | [35] |
| 62 | ARLGCPVIP | 1.37 ± 0.33 | [35] |
| 63 | SEGGCPLIL | 3.75 ± 0.77 | [35, 54] |
| 64 | TPILCPFHL | 0.60 ± 0.41 | [35] |
| 65 | AAAACPAAA | 4.73 ± 1.83 | [35] |
| 66 | SSIPCLHYK | 0.81 ± 0.51 | [35] |
| 67 | DESACPYVM | 3.27 ± 1.44 | [35] |
| 68 | AIRRCSTFQ | 0.50 ± 0.20 | [81] |
| 69 | HELVCAAST | 0.40 ± 0.19 | [81] |
| 70 | QKGVCQNTG | 2.25 ± 0.70 | [81] |
| 71 | AAHYHTYER | 0.83 ± 0.33 | [82] |
| 72 | FKAAHKHVR | 0.99 ± 0.21 | [82] |
| 73 | FLFYHSQSG | 4.48 ± 2.20 | [54] |
| *B, norleucine as substituent for methionine; ^†^these K_D_ values have been reevaluated | | | |

**References**

1. Lathrop JT, Timko MP. Regulation by heme of mitochondrial protein transport through a conserved amino acid motif. Adv Sci. 1993;259:522–6.

2. Munakata H, Sun JY, Yoshida K, Nakatani T, Honda E, Hayakawa S, et al. Role of the heme regulatory motif in the heme-mediated inhibition of mitochondrial import of 5-aminolevulinate synthase. J Biochem. 2004;136:233–8.

3. Kubota Y, Nomura K, Katoh Y, Yamashita R, Kaneko K, Furuyama K. Novel mechanisms for heme-dependent degradation of ALAS1 protein as a component of negative feedback regulation of heme biosynthesis. J Biol Chem. 2016;291:20516–29.

4. Goodfellow BJ, Dias JS, Ferreira GC, Henklein P, Wray V, Macedo AL. The solution structure and heme binding of the presequence of murine 5-aminolevulinate synthase. FEBS Lett. 2001;505:325–31.

5. Atamna H, Frey WH. A role for heme in Alzheimer’s disease: Heme binds amyloid β and has altered metabolism. Proc Natl Acad Sci U S A. 2004;101:11153–8.

6. Atamna H, Frey WH, Ko N. Human and rodent amyloid-β peptides differentially bind heme: Relevance to the human susceptibility to Alzheimer’s disease. Arch Biochem Biophys. 2009;487:59–65.

7. Pramanik D, Dey SG. Active site environment of heme-bound amyloid β peptide associated with Alzheimers Disease. J Am Chem Soc. 2011;133:81–7.

8. Zhou Y, Wang J, Liu L, Wang R, Lai X, Xu M. Interaction between amyloid-β peptide and heme probed by electrochemistry and atomic force microscopy. ACS Chem Neurosci. 2013;4:535–9.

9. Wißbrock A, Kühl T, Silbermann K, Becker AJ, Ohlenschläger O, Imhof D. Synthesis and evaluation of amyloid β derived and amyloid β independent enhancers of the peroxidase-like activity of heme. J Med Chem. 2017;60:373–85.

10. Hu R-G, Wang H, Xia Z, Varshavsky A. The N-end rule pathway is a sensor of heme. Proc Natl Acad Sci U S A. 2008;105:76–81.

11. Yang F, Xia X, Lei HY, Wang ED. Hemin binds to human cytoplasmic arginyl-tRNA synthetase and inhibits its catalytic activity. J Biol Chem. 2010;285:39437–46.

12. Ogawa K, Sun J, Taketani S, Nakajima O, Nishitani C, Sassa S, et al. Heme mediates derepression of Maf recognition element through direct binding to transcription repressor Bach1. EMBO J. 2001;20:2835–43.

13. Hira S, Tomita T, Matsui T, Igarashi K, Ikeda-Saito M. Bach1, a heme-dependent transcription factor, reveals presence of multiple heme binding sites with distinct coordination structure. IUBMB Life. 2007;59:542–51.

14. Zenke-Kawasaki Y, Dohi Y, Katoh Y, Ikura T, Ikura M, Asahara T, et al. Heme induces ubiquitination and degradation of the transcription factor Bach1. Mol Cell Biol. 2007;27:6962–71.

15. Segawa K, Watanabe-Matsui M, Matsui T, Igarashi K, Murayama K. Functional heme binding to the intrinsically disordered C-terminal region of bach1, a transcriptional repressor. Tohoku J Exp Med. 2018;247:153–9.

16. Watanabe-Matsui M, Muto A, Matsui T, Itoh-Nakadai A, Nakajima O, Murayama K, et al. Heme regulates B-cell differentiation, antibody class switch, and heme oxygenase-1 expression in B cells as a ligand of Bach2. Blood. 2011;117:5438–48.

17. Watanabe-Matsui M, Matsumoto T, Matsui T, Ikeda-Saito M, Muto A, Murayama K, et al. Heme binds to an intrinsically disordered region of Bach2 and alters its conformation. Arch Biochem Biophys. 2015;565:25–31.

18. Suenaga T, Watanabe-Matsui M, Uejima T, Shima H, Matsui T, Ikeda-Saito M, et al. Charge-state-distribution analysis of Bach2 intrinsically disordered heme binding region. J Biochem. 2016;160:291–8.

19. Tang XD, Xu R, Reynolds MF, Garcia ML, Heinemann SH, Hoshi T. Haem can bind to and inhibit mammalian calcium-dependent Slo1 BK channels. Nature. 2003;425:531–5.

20. Williams SEJ, Wootton P, Mason HS, Bould J, Iles DE, Riccardi D, et al. Hemoxygenase-2 is an oxygen sensor for a calcium-sensitive potassium channel. Science (80- ). 2004;306:2093–7.

21. Horrigan FT, Heinemann SH, Hoshi T. Heme regulates allosteric activation of the Slo1 BK channel. J Gen Physiol. 2005;126:7–21.

22. Jaggar JH, Li A, Parfenova H, Liu J, Umstot ES, Dopico AM, et al. Heme is a carbon monoxide receptor for large-conductance Ca 2+-activated K+ channels. Circ Res. 2005;97:805–12.

23. Yi L, Morgan JT, Ragsdale SW. Identification of a thiol/disulfide redox switch in the human BK channel that controls its affinity for heme and CO. J Biol Chem. 2010;285:20117–27.

24. Dimitrov JD, Roumenina LT, Doltchinkova VR, Vassilev TL. Iron ions and haeme modulate the binding properties of complement subcomponent C1q and of immunoglobulins. Scand J Immunol. 2007;65:230–9.

25. Roumenina LT, Radanova M, Atanasov BP, Popov KT, Kaveri S V., Lacroix-Desmazes S, et al. Heme interacts with C1q and inhibits the classical complement pathway. J Biol Chem. 2011;286:16459–69.

26. Frimat M, Tabarin F, Dimitrov JD, Poitou C, Halbwachs-Mecarelli L, Fremeaux-Bacchi V, et al. Complement activation by heme as a secondary hit for atypical hemolytic uremic syndrome. Blood. 2013;122:282–92.

27. Lukat-Rodgers GS, Correia C, Botuyan MV, Mer G, Rodgers KR. Heme-based Sensing by the Mammalian Circadian Protein, CLOCK. Inorg Chem. 2010;49:6349–65.

28. Meier M, Janosik M, Kery V, Kraus JP, Burkhard P. Structure of human cystathionine β-synthase: A unique pyridoxal 5′-phosphate-dependent heme protein. EMBO J. 2001;20:3910–6.

29. Taoka S, Lepore BW, Kabil Ö, Ojha S, Ringe D, Banerjee R. Human cystathionine β-synthase is a heme sensor protein. Evidence that the redox sensor is heme and not the vicinal cysteines in the CXXC motif seen in the crystal structure of the truncated enzyme. Biochemistry. 2002;41:10454–61.

30. Weeks CL, Singh S, Madzelan P, Banerjee R, Spiro TG. Heme regulation of human cystathionine β-synthase activity: Insights from fluorescence and Raman spectroscopy. J Am Chem Soc. 2009;131:12809–16.

31. Kumar A, Wißbrock A, Goradia N, Bellstedt P, Ramachandran R, Imhof D, et al. Heme interaction of the intrinsically disordered N-terminal peptide segment of human cystathionine-β-synthase. Sci Rep. 2018;8:1–9.

32. Faller M, Matsunaga M, Yin S, Loo JA, Guo F. Heme is involved in microRNA processing. Nat Struct Mol Biol. 2007;14:23–9.

33. Barr I, Smith AT, Senturia R, Chen Y, Scheidemantle BD, Burstyn JN, et al. DiGeorge Critical Region 8 (DGCR8) is a double-cysteine-ligated heme protein. J Biol Chem. 2011;286:16716–25.

34. Weitz SH, Gong M, Barr I, Weiss S, Guo F. Processing of microRNA primary transcripts requires heme in mammalian cells. Proc Natl Acad Sci. 2014;111:1861–6.

35. Kühl T, Wißbrock A, Goradia N, Sahoo N, Galler K, Neugebauer U, et al. Analysis of Fe(III) heme binding to cysteine-containing heme-regulatory motifs in proteins. ACS Chem Biol. 2013;8:1785–93.

36. Mense SM, Zhang L. Heme: a versatile signaling molecule controlling the activities of diverse regulators ranging from transcription factors to MAP kinases. Cell Res. 2006;16:681–92.

37. Miksanova M, Igarashi J, Minami M, Sagami I, Yamauchi S, Kurokawa H, et al. Characterization of heme-regulated eIF2α kinase: Roles of the N-terminal domain in the oligomeric state, heme binding, catalysis, and inhibition. Biochemistry. 2006;45:9894–905.

38. Igarashi K, Murase M, Iizuka A, Pichierri F, Martinkova M, Shimizu T, et al. Elucidation of the heme binding site of heme-regulated eukaryotic initiation factor 2α kinase and the role of the regulatory motif in heme sensing by spectroscopic and catalytic studies of mutant proteins. J Biol Chem. 2008;283:18782–91.

39. Green D, Furby FH, Berndt MC. The interaction of the VIII/von Willebrand factor complex with hematin. Thromb Haemost. 1986;56:277–82.

40. Repessé Y, Dimitrov JD, Peyron I, Moshai EF, Kiger L, Dasgupta S, et al. Heme binds to factor VIII and inhibits its interaction with activated factor IX. J Thromb Haemost. 2012;10:1062–71.

41. Orino K. Functional binding analysis of human fibrinogen as an iron- and heme-binding protein. BioMetals. 2013;26:789–94.

42. Ke Z, Huang Q. Haem-assisted dityrosine-cross-linking of fibrinogen under non-thermal plasma exposure: One important mechanism of facilitated blood coagulation. Sci Rep. 2016;6 May:1–8. doi:10.1038/srep26982.

43. Grdisa M, White MK. Expression of glyceraldehyde-3-phosphate dehydrogenase during differentiation of HD3 cells. Eur J Cell Biol. 1996;71:177–82.

44. Campanale N, Nickel C, Daubenberger CA, Wehlan DA, Gorman JJ, Klonis N, et al. Identification and characterization of heme-interacting proteins in the malaria parasite, Plasmodium falciparum. J Biol Chem. 2003;278:27354–61.

45. Famin O, Ginsburg H. The treatment of Plasmodium falciparum -infected erythrocytes with chloroquine leads to accumulation of ferriprotoporphyrin IX bound to particular parasite proteins and to the inhibition of the parasite’s 6-phosphogluconate dehydrogenase . Parasite. 2003;10:39–50.

46. Chakravarti R, Aulak KS, Fox PL, Stuehr DJ. GAPDH regulates cellular heme insertion into inducible nitric oxide synthase. Proc Natl Acad Sci. 2010;107:18004–9.

47. Hannibal L, Collins D, Brassard J, Chakravarti R, Vempati R, Dorlet P, et al. Heme binding properties of glyceraldehyde-3-phosphate dehydrogenase. Biochemistry. 2012;51:8514–29.

48. McCoubrey WK, Huang TJ, Maines MD. Heme oxygenase-2 is a hemoprotein and binds heme through heme regulatory motifs that are not involved in heme catalysis. J Biol Chem. 1997;272:12568–74.

49. Yi L, Ragsdale SW. Evidence that the heme regulatory motifs in heme oxygenase-2 serve as a thiol/disulfide redox switch regulating heme binding. J Biol Chem. 2007;282:21056–67.

50. Yi L, Jenkins PM, Leichert LI, Jakob U, Martens JR, Ragsdale SW. Heme regulatory motifs in heme oxygenase-2 form a thiol/disulfide redox switch that responds to the cellular redox state. J Biol Chem. 2009;284:20556–61.

51. Fleischhacker AS, Sharma A, Choi M, Spencer AM, Bagai I, Hoffman BM, et al. The C-terminal heme regulatory motifs of heme oxygenase-2 are redox-regulated heme binding sites. Biochemistry. 2015;54:2709–18.

52. Yang J, Kim KD, Lucas A, Drahos KE, Santos CS, Mury SP, et al. A novel heme-regulatory motif mediates heme-dependent degradation of the circadian factor Period 2. Mol Cell Biol. 2008;28:4697–711.

53. Dimitrov JD, Roumenina LT, Doltchinkova VR, Mihaylova NM, Lacroix-Desmazes S, Kaveri S V., et al. Antibodies use heme as a cofactor to extend their pathogen elimination activity and to acquire new effector functions. J Biol Chem. 2007;282:26696–706.

54. Wißbrock A, Goradia NB, Kumar A, Paul George AA, Kühl T, Bellstedt P, et al. Structural insights into heme binding to IL-36α proinflammatory cytokine. Sci Rep. 2019;9:16893.

55. Ogura M, Endo R, Ishikawa H, Takeda Y, Uchida T, Iwai K, et al. Redox-dependent axial ligand replacement and its functional significance in heme-bound iron regulatory proteins. J Inorg Biochem. 2018;182:238–48. doi:10.1016/j.jinorgbio.2018.01.007.

56. Nishitani Y, Okutani H, Takeda Y, Uchida T, Iwai K, Ishimori K. Specific heme binding to heme regulatory motifs in iron regulatory proteins and its functional significance. J Inorg Biochem. 2019;198 June:110726. doi:10.1016/j.jinorgbio.2019.110726.

57. Yamanaka K, Ishikawa H, Megumi Y, Tokunaga F, Kanie M, Rouault TA, et al. Identification of the ubiquitin-protein ligase that recognizes oxidized IRP2. Nat Cell Biol. 2003;5:336–40.

58. Ishikawa H, Kato M, Hori H, Ishimori K, Kirisako T, Tokunaga F, et al. Involvement of heme regulatory motif in heme-mediated ubiquitination and degradation of IRP2. Mol Cell. 2005;19:171–81.

59. Yao X, Balamurugan P, Arvey A, Leslie C, Zhang L. Heme controls the regulation of protein tyrosine kinases Jak2 and Src. Biochem Biophys Res Commun. 2010.

60. Burton MJ, Kapetanaki SM, Chernova T, Jamieson AG, Dorlet P, Santolini J, et al. A heme-binding domain controls regulation of ATP-dependent potassium channels. Proc Natl Acad Sci. 2016;113:3785–90.

61. Kimura I, Nakayama Y, Yamauchi H, Konishi M, Miyake A, Mori M, et al. Neurotrophic activity of neudesin, a novel extracellular heme-binding protein, is dependent on the binding of heme to its cytochrome b 5-like heme/steroid-binding domain. J Biol Chem. 2008;283:4323–31.

62. Kimura I, Nakayama Y, Konishi M, Kobayashi T, Mori M, Ito M, et al. Neuferricin, a novel extracellular heme-binding protein, promotes neurogenesis. J Neurochem. 2010;112:1156–67.

63. Chernova T, Steinert JR, Guerin CJ, Nicotera P, Forsythe ID, Smith AG. Neurite Degeneration Induced by Heme Deficiency Mediated via Inhibition of NMDA Receptor-Dependent Extracellular Signal-Regulated Kinase 1/2 Activation. J Neurosci. 2007;27:8475–85.

64. Kannan M, Steinert JR, Forsythe ID, Smith AG, Chernova T. Mevastatin accelerates loss of synaptic proteins and neurite degeneration in aging cortical neurons in a heme-independent manner. Neurobiol Aging. 2010;31:1543–53. doi:10.1016/j.neurobiolaging.2008.09.004.

65. Shen J, Sheng X, Chang ZN, Wu Q, Wang S, Xuan Z, et al. Iron metabolism regulates p53 signaling through direct Heme-p53 interaction and modulation of p53 localization, stability, and function. Cell Rep. 2014;7:180–93. doi:10.1016/j.celrep.2014.02.042.

66. Shen J, Sheng X, Chang ZN, Wu Q, Xie D, Wang F, et al. The heme–p53 interaction: Linking iron metabolism to p53 signaling and tumorigenesis. Mol Cell Oncol. 2016;3:5–7.

67. Min L, Strushkevich N V., Harnastai IN, Iwamoto H, Gilep AA, Takemori H, et al. Molecular identification of adrenal inner zone antigen as a heme-binding protein. FEBS J. 2005;272:5832–43.

68. Kabe Y, Nakane T, Koike I, Yamamoto T, Sugiura Y, Harada E, et al. Haem-dependent dimerization of PGRMC1/Sigma-2 receptor facilitates cancer proliferation and chemoresistance. Nat Commun. 2016;7 May 2015:1–13. doi:10.1038/ncomms11030.

69. Yin L, Wu N, Curtin JC, Qatanani M, Szwergold NR, Reid RA, et al. Rev-erbα, a heme sensor that coordinates metabolic and circadian pathways. Science (80- ). 2007;318:1786–9.

70. Raghuram S, Stayrook KR, Huang P, Rogers PM, Nosie AK, McClure DB, et al. Identification of heme as the ligand for the orphan nuclear receptors REV-ERBα and REV-ERBβ. Nat Struct Mol Biol. 2007;14:1207–13.

71. Marvin KA, Reinking JL, Lee AJ, Pardee K, Krause HM, Burstyn JN. Nuclear receptors Homo sapiens rev-erbβ and Drosophila melanogaster E75 are thiolate-ligated heme proteins which undergo redox-mediated ligand switching and bind CO and NO. Biochemistry. 2009;48:7056–71.

72. Pardee KI, Xu X, Reinking J, Schuetz A, Dong A, Liu S, et al. The structural basis of gas-responsive transcription by the human nuclear hormone receptor REV-ERBβ. PLoS Biol. 2009;7:0384–98.

73. Gupta N, Ragsdale SW. Thiol-disulfide redox dependence of heme binding and heme ligand switching in nuclear hormone receptor rev-erbβ. J Biol Chem. 2011;286:4392–403.

74. Carter EL, Gupta N, Ragsdale SW. High affinity heme binding to a heme regulatory motif on the nuclear receptor rev-erbβ leads to its degradation and indirectly regulates its interaction with nuclear receptor corepressor. J Biol Chem. 2016;291:2196–222.

75. Carter EL, Ramirez Y, Ragsdale SW. The heme-regulatory motif of nuclear receptor Rev-erbβ is a key mediator of heme and redox signaling in circadian rhythm maintenance and metabolism. J Biol Chem. 2017;292:11280–99.

76. Westberg JA, Jiang J, Andersson LC. Stanniocalcin 1 binds hemin through a partially conserved heme regulatory motif. Biochem Biophys Res Commun. 2011;409:266–9. doi:10.1016/j.bbrc.2011.05.002.

77. Jiang J, Westberg JA, Andersson LC. Stanniocalcin 2, forms a complex with heme oxygenase 1, binds hemin and is a heat shock protein. Biochem Biophys Res Commun. 2012;421:274–9. doi:10.1016/j.bbrc.2012.03.151.

78. Figueiredo RT, Fernandez PL, Mourao-Sa DS, Porto BN, Dutra FF, Alves LS, et al. Characterization of heme as activator of toll-like receptor 4. J Biol Chem. 2007;282:20221–9.

79. Wakasugi K. Human tryptophanyl-tRNA synthetase binds with heme to enhance its aminoacylation activity. Biochemistry. 2007;46:11291–8.

80. Nikolajski M, Kühl T, Heinemann SH, Schlott B, Imhof D, Sahoo N. Determination of Hemin-Binding Characteristics of Proteins by a Combinatorial Peptide Library Approach. ChemBioChem. 2011;12:2846–55.

81. Brewitz HH, Kühl T, Goradia N, Galler K, Popp J, Neugebauer U, et al. Role of the Chemical Environment beyond the Coordination Site: Structural Insight into FeIII Protoporphyrin Binding to Cysteine-Based Heme-Regulatory Protein Motifs. ChemBioChem. 2015.

82. Brewitz HH, Goradia N, Schubert E, Galler K, Kühl T, Syllwasschy BF, et al. Heme interacts with histidine-and tyrosine-based protein motifs and inhibits enzymatic activity of chloramphenicol acetyltransferase from Escherichia coli. Biochim Biophys Acta (BBA)-General Subj. 2016;1860:1343–53.

83. Schubert E, Florin N, Duthie F, Brewitz HH, Kühl T, Imhof D, et al. Spectroscopic studies on peptides and proteins with cysteine-containing heme regulatory motifs (HRM). J Inorg Biochem. 2015;148:49–56.
